# Robust and Cooperative Localization for Underwater Sensor Networks in the Existence of Malicious Anchors

**DOI:** 10.3390/s19204519

**Published:** 2019-10-17

**Authors:** Wenyu Cai, Junlei Yang, Meiyan Zhang, Shiling Peng, Junyi Yang

**Affiliations:** 1 College of Electronics and Information, Hangzhou Dianzi University, Hangzhou 310018, China; yangjunlei0@163.com (J.Y.); psl@hdu.edu.cn (S.P.); 2 Zhejiang Provincial Key Lab of Equipment Electronics, Hangzhou Dianzi University, Hangzhou 310018, China; 3 School of Electrical Engineering, Zhejiang University of Water Resources and Electric Power, Hangzhou 310018, China; 4 Marine Engineering Center, Hangzhou Dianzi University, Hangzhou 310018, China

**Keywords:** underwater sensor networks, robust localization, cooperative localization, malicious anchor nodes, reputation voting

## Abstract

Precise and robust localization in three-dimensional underwater sensor networks is still an important research problem. This problem is particularly challenging if there are some malicious anchors among ordinary anchor nodes that will broadcast their locations falsely and deliberately. In this paper, we study how to self-localize large teams of underwater sensor nodes under the condition that some malicious anchor nodes mixed with ordinary anchors. Due to malicious characteristic of some deliberate anchor nodes, an iterative and cooperative 3D-localization algorithm for underwater sensor networks in the existence of malicious anchors is proposed in this paper. The proposed robust localization algorithm takes advantage of distributed reputation voting method within 1-Hop neighboring reference nodes to detect and eliminate malicious anchor nodes. Moreover, one kind of Minimum Mean Squared Error estimation based iterative localization method is applied to determine accurate location information. Additionally, we analyze and prove that our localization algorithm would have a bounded error when the number of malicious anchors is smaller than a certain threshold. Extensive simulation results are provided to demonstrate performance improvements comparing to traditional Minimum Mean Squared Error and Attack Resistant Minimum Mean Squared Error based localization methods in terms of localization accuracy and coverage ratio.

## 1. Introduction

Localization in Underwater Sensor Networks have attracted significant interests in recent years [1]. Position information accompanying with sensory data are vital to many monitoring activities in sensor networks. For example, in environmental monitoring applications, it is necessary to know the specific regional position corresponding to the collected environmental information, which is the basis on further measures and decisions. Currently, the simplest and most straightforward way to determine sensor node’s location is of course using the Global Positioning System (GPS). However, GPS is unsuitable to use in underwater environments [2]. Compared to radio wave, sound wave has good propagation characteristics in water, so it has become a more suitable underwater communication carrier. Although traditional relative distance estimation methods, such as ToA (Time of Arrival) or TDoA (Time Difference of Arrival), would suffer from severe multi-path propagation and Doppler effects in underwater acoustic channel, underwater localization system generally follows acoustic communication and measurement approach. As we all know, Ultra-Short BaseLine (USBL) and Doppler Velocity Logs (DVL) are too costly to install on all underwater nodes, therefore, it is no guaranteed that all sensor nodes can locate themselves. Moreover, underwater nodes are often deployed sparsely because of their high costs, so direct communication from anchor nodes to ordinary nodes may not be available, resulting in lower localization coverage. Consequently, some underwater nodes may lack of the required number of reference nodes within their communication range to aid localization.

Numerous localization methods [3,4] have been proposed for UWSN in recent years. RSS-based underwater localization algorithms are discussed in [5,6], which determine the location of an unknown normal sensor from a certain measurement set of potential anchor nodes. However, RSS-based localization suffers from harsh underwater environments, such as measurement noises, heavily. In recent years, there have been many studies on the localization of underwater sensor networks. The problem of three-dimensional localization of underwater sensor networks with unknown water currents using only range measurements is investigated in [7]. This scheme applies the rigidity theory and maintains a virtual rigid structure through projection. Since underwater nodes move constantly with ocean currents and measurement noises vary with distances, a novel beacon-free algorithm is proposed in [8] considering additional challenges posed by harsh underwater environments. Recursive Location Estimation (RLE) algorithm [9], a hierarchical localization scheme for stationary underwater acoustic sensor networks, uses an extended Euclidean distance estimation to determine its distance measurement. RLE algorithm performs well in dense underwater sensor networks, but it suffers from low localization coverage in sparse networks. Moreover, localization error will propagate when the distributed RLE algorithm is used in large-scale networks, and impacts by measurement error on localization accuracy of RLE algorithm have been analyzed in [10].

Nevertheless, in many hostile environments, there may be malicious attacks to mislead the location estimation of sensor nodes, so the positioning process is highly vulnerable to malicious attacks from some enemies intentionally [11]. As a result, anchor nodes are at higher risk of being caught at any time. Malicious anchor nodes formed after being captured can initiate multiple attacks, giving wrong location information and threatening the security of localization system seriously. Many existing localization methods become vulnerable in such attacks. Authentication can provide some, but limited, reliability through cryptography. Even with encrypted location references, it is still possible for attackers to compromise some anchor nodes or simply replace location references intercepted at different locations. Therefore, in the model studied of this paper, we assume that there are *k* malicious anchor nodes among these *m* anchor sets.

In the past, researchers have followed two approaches towards overcoming the problem of malicious nodes in localization algorithms [12]. One is to find inconsistencies in the communication process of sensor nodes and eliminate them before localization. The other is to minimize the position error in the presence of malicious nodes by certain algorithms, so as tolerate the interference by malicious nodes. Pires et al. [13] propose a method to identify malicious nodes by detecting the signal strength of malicious message transmissions in sensor networks. In [14], an attack detection module is proposed for detection boundary error. The damaged anchor nodes can be detected by this secure localization module, but it is only suitable to single-hop positioning. Curiac et al. [15] propose a neural network and an autoregressive model to estimate output values from sensor nodes, and then compares and obtains the difference, so as to determine which malicious nodes are. Later, they propose malicious node self-destruction algorithm in [16]. Wei et al. [17] propose two centralized node position verification algorithms named GFM (Greedy Filtering by Matrix) and TI (Trustability Indicator). However, both algorithms must collect location information of all nodes and corresponding observations to verify positioning results. Generally speaking, the robustness of these algorithms are poor, and attackers can easily tamper with neighboring observation and interference detection algorithm. Du et al. [18] give a Localization Anomaly Detection (LAD) scheme to detect abnormal anchor nodes during positioning process. The LAD scheme relies on the distribution information of sensor nodes. If the accurate distribution probability cannot be obtained, the LAD detection result will be greatly affected. Moreover, the LAD scheme only stays in the detection phase of the anomaly, but does not give how to handle the exception and how to improve localization accuracy after anomalies are discovered. Moore et al. [19] describe a distributed, linear-time algorithm for localizing sensor nodes in the presence of range measurement noise. They formulate the localization problem as a two-dimensional graph realization problem. Li et al. [20] explore robust statistical methods to make localization attack-tolerant. The advantage is the least squares computation when there is no attack and it will switch to the robust mode when attacked. However, this method is only suitable for triangulation and RF-based fingerprints based localization. Buchegger et al. [21] propose CONFIDANT with predetermined trust measurement. They cope with the localization performance by retaliating for malicious behavior and warning affiliated nodes. Anchor nodes learn not only from their own experience, but also from observing their neighborhood. In [22], a probabilistic nested packet marking method (PNM) is proposed. In order to protect the upstream nodes from marking on data packets, each forwarding node marks data packets in a nested manner, preventing the collusion node from covering the data packet. However, as anchor nodes are continuously marked cryptographically, packets will become larger and larger and thus increase traffic overhead significantly. However, these algorithms do not involve cooperation between anchor nodes, and thus severely limit the performance to detect compromised anchors. Attack-resistant minimum mean square error (ARMMSE) [23] is a greedy algorithm based on an iterative Least Trimmed Squares (LTS) approach to identify and eliminate malicious anchors one by one. In [24], two methods for robust localization in the presence of malicious anchor nodes are studied. The first method filters out malicious beacon signals on the basis of the consistency among multiple beacon signals. The second method tolerates malicious beacon signals by adopting an iteratively refined voting scheme. The disadvantage is that the greedy algorithm has to be used to obtain an approximate solution due to the explosion of combination number. But grid size and number will affect localization performance, such as solution time. Srinivasan et al. [25] propose an anchor trust scheme based on distributed reputation mechanism for excluding malicious anchor nodes that provide false location information. This algorithm is simple, but the robustness and accuracy still need to be improved. The above documents all propose various methods for eliminating or ignoring malicious nodes for positioning, but due to the complexity of underwater environment and limitations of each algorithm, most of them are only applicable for two-dimensional environment. In the underwater environment, sensor nodes are deployed randomly in particular three-dimensional space. However, the performance may be reduced significantly if just apply general localization algorithms of two-dimensional space to three-dimensional space directly. Therefore the positioning in three-dimensional underwater sensor networks faces great challenges and so it is very crucial for underwater monitoring system.

Moreover, there are some researches for eliminating malicious nodes in recent years. Liu et al. [26] present a suite of methods to detect malicious beacon signals and identify malicious anchors, so avoid false detection and revoke malicious anchors. Their revocation scheme works on the basis of two counters maintained in each anchor node. The concept of weighted trust proposed in [27] determines the ratio between data transmitted by sensor node and the final fusion result. This method has a certain detection ratio for detecting malicious nodes with lower complexity. However, when the number of malicious nodes is large, the fusion result and the weight of sensor node will be greatly affected, which is prone to produce false positives (no malicious nodes detected) and false negative detection (normal nodes are incorrectly detected as malicious nodes). Ganeriwal et al. [28] investigate an approach to allow sensor nodes to develop a community of trust. In their framework, each sensor node maintains reputation metrics which both represent past behavior of other nodes and inherent aspect in predicting future behavior. Based on the reputation-based authentication model proposed in [29], the literature [30] proposes an entity authentication model based on reputation and trust groups. However, this scheme is only on basis of trusted authentication of the key group. If the malicious node obtains the key, the resilience of this model will be greatly reduced.

Referring to the idea of confidence value and weight in the above literature, and considering the characteristics and difficulties of three-dimensional underwater localization, we propose a robust and cooperative 3D-localization algorithm for underwater sensor networks in the existence of some malicious anchor nodes. The proposed localization algorithm takes advantage of distributed reputation voting method within one-hop neighboring reference nodes to detect and remove malicious anchors. Moreover, position-unknown sensor nodes use MMSE (Minimum Mean Squared Error) [9] based iterative positioning algorithm to make the just positioned sensor node become new reference node, thus help to localize other sensor nodes that do not know their locations.

*Statement of Contributions*: The main contributions of this paper are summarized as follows: (1) Providing a reputation voting based malicious anchors detection and elimination mechanism within 1-Hop neighborhood; and (2) design an iterative and distributed 3D localization algorithm in the existence of malicious anchors. To the best of our knowledge, this is the first report to design robust and iterative localization for three-dimensional underwater sensor networks in the existence of malicious anchor nodes.

The rest of this paper is organized as follows. Section 2 presents preliminaries and problem statement. In Section 3, a distributed and iterative localization method for UWSN to overcome the impacts by malicious anchors is presented in detail. Extensive simulation results to verify the robustness of our localization algorithm are shown in Section 4, followed by the conclusion in Section 5.

## 2. Preliminaries and Problem Statement

**Figure 1:**
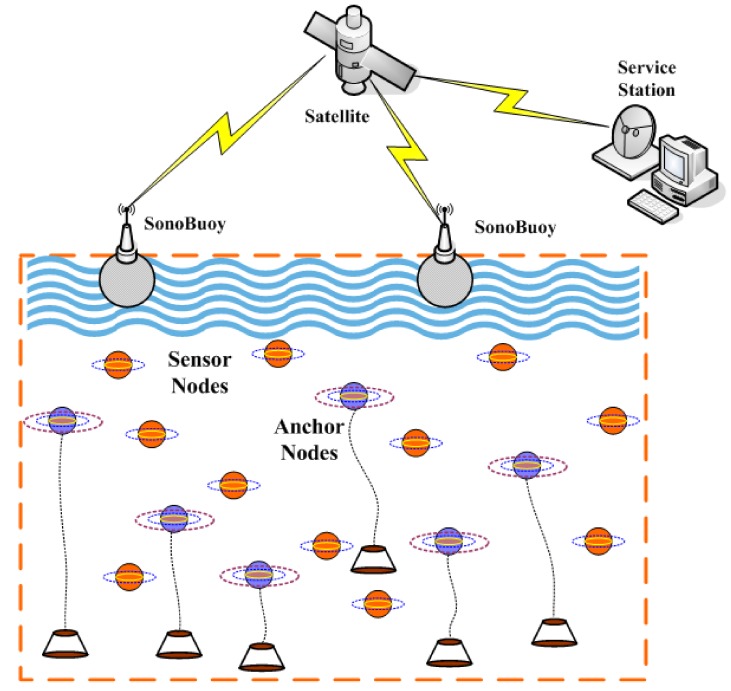
Localization topology of three-dimensional underwater sensor networks.

As depicted in Figure 1, there are *n* sensor nodes $\left(S_{i},i=1,2,\cdots ,n\right)$ (the brown ball) to be localized and *m* anchor nodes $\left(A_{j},j=1,2,\cdots ,m\right)$ (the blue ball) with known positions $a_{j}=\left[x_{j},y_{j},z_{j}\right]\in R^{3}$ deployed randomly in a W×W×W cubic underwater space. With a multi-hop transmission manner, sensor nodes have the ability to transmit information to any SonyBuoy via acoustic communication, while surface SonoBuoy can transmit collected data to Service Station through Satellite or RF communication finally. It is assumed that powerful and immobile anchor nodes can get their positions with USBL (Ultra-Short BaseLine), but ordinary sensor nodes communicate only with its 1-Hop neighbors hence they can be localized with the help of its neighboring reference nodes, which include anchor nodes and sensor nodes with known locations already. On the basis of actual employment situation of underwater sensor networks, the maximal transmission ranges of sensor nodes and anchor nodes are similar usually, herein they are set to the same threshold value *R*. In addition, compared to vast underwater monitoring region, the drifting movement of underwater sensor nodes by oceanic current can be ignored. As a result, the proposed localization algorithm only needs to be executed once or at fixed intervals. For simplicity, we assume that the IDs of sensor nodes employ both software and hardware encryption to enhance security, so they could not be tampered by malicious anchors easily.

Generally speaking, anchor nodes represent those sensor nodes with locations that are known prior to the localization process, and it is assumed that the position information provided by anchor nodes is correct and accurate. However, in hostile environments, there may be some malicious attacks in order to sabotage or mislead the localization process of sensor nodes. Unlike existing assumptions, we suppose that *k* anchor nodes within *m* anchor nodes are malicious anchor nodes, and others are honest anchor nodes. Let the set of honest anchors and malicious anchors be denoted by H and M, respectively. That means M⊆A, H⊆A, M⋃H=A. It is assumed that malicious anchor nodes will broadcast their locations falsely and thus pose negative effect on localization accuracy and robustness. Besides, malicious anchors percent *p* is defined as the ratio between the number of malicious nodes and that of anchor nodes, which is $p=\frac{k}{m}$. It is important to note that *k* or *p* is not necessarily known to any underwater sensors or anchor nodes. Moreover, we assume that the identity number of each anchor node is unique and cannot be maliciously tampered easily.

In order to illustrate the detailed methodology of proposed algorithm, we summarize the simplified notations in Table 1 for convenience.

Regardless of being honest or dishonest, each anchor node $A_{j}$ provides a measurement of the distance between sensor nodes and itself. In the case of anchor node $A_{j}$ is honest, the difference between the estimated and actual distance is assumed to be very small, i.e., $\left|\tilde{dst}\left(S_{i},A_{j}\right)-dst\left(S_{i},A_{j}\right)\right|<\epsilon ,\forall S_{i}\in S$, where ϵ is a small constant. Otherwise, the above formula does not always hold when $A_{j}$ is a malicious anchor node. The precise distance between $S_{i}$ and $S_{j}$ is the Euclidean distance between position coordinates of $s_{i}$ and $s_{j}$, which is denoted by $\|s_{i}-s_{j}\|$. The operator ‖‖ denotes the distance calculation between two coordinates in $R^{3}$. The distance measurements between sensor nodes and anchor nodes are defined as $\tilde{dst}\left(S_{i},A_{j}\right)$, which are assumed to be random variables that follow some fixed probability distributions due to noise and interference among sensor nodes.

(1) $$\begin{array}{c} \tilde{dst}\left(S_{i},A_{j}\right)=\left\|s_{i}-a_{j}\right\|+\mu_{ij},\forall A_{j}\in H. \end{array}$$

For each anchor node $A_{j}\in M$, $\tilde{dst}\left(S_{i},A_{j}\right)$ is a value selected arbitrarily by the adversary. In other words, the above equation will not always necessarily be hold.

(2) $$\begin{array}{c} \tilde{dst}\left(S_{i},A_{j}\right)\neq \left\|s_{i}-a_{j}\right\|+\mu_{ij},\forall A_{j}\in M, \end{array}$$

where $\mu_{ij}$ s are i.i.d. Gaussian random variables with zero mean and variance of $\sigma^{2}$. For honest anchor nodes, there are $E\left(\tilde{dst}\left(S_{i},A_{j}\right)\right)=\left\|s_{i}-a_{j}\right\|$. Ideally, this difference should be zero when the anchor node is honest, but such discrepancies in distance estimates can occur due to measurement errors. That is to say, the expected value of the estimated distance $\tilde{dst}\left(S_{i},A_{j}\right)$ for each anchor $A_{j}\in H$ is the trustworthy distance between anchor nodes $S_{i}$ and $A_{j}$. For malicious anchor nodes, $E\left(\tilde{dst}\left(S_{i},A_{j}\right)\right)\neq \left\|s_{i}-a_{j}\right\|$. In other words, malicious anchor will cause measurement outliers so that it cannot produce trustworthy position estimates.

Let δ be the average localization error of all sensor nodes in underwater sensor networks, which is defined as the Euclidean distance between actual position and the one output by localization algorithm. $s_{i}$ and $\tilde{s_{i}}$ denote actual coordinates and measured coordinates of sensor nodes $S_{i}$, respectively. Therefore, the optimization objective of robust localization for underwater sensor networks in the existence of malicious anchors is given as,

Objective:

(3) $$\begin{array}{cc} \mathrm{minimize}\; & \delta =\frac{1}{n}\cdot \sum_{i=1}^{n}\left\|s_{i}-\tilde{s_{i}}\right\|, \end{array}$$

subject to:

(4) $$\begin{array}{cc} \tilde{dst}\left(S_{i},A_{j}\right) & =\|s_{i}-a_{j}\|+\mu_{ij},\forall A_{j}\in H \end{array}$$

(5) $$\begin{array}{cc} \tilde{dst}\left(S_{i},A_{j}\right) & =\|s_{i}-a_{j}\|+\zeta +\mu_{ij},\forall A_{j}\in M, \end{array}$$

where $\tilde{dst}\left(S_{i},A_{j}\right)$ denotes the distance measurements between sensor node $S_{i}$ and anchor node $A_{j}$, and ζ denotes certain inconsistent malicious degree caused by malicious anchors. Equation (3) is our objective function representing the average localization error of all available sensor nodes for localization. The constraints (4), (5) denote bounded error and unbounded error caused by honest anchors and malicious anchors, respectively. In other words, each underwater sensor nodes wants to compute its own location using distance estimates with reference nodes that know their own locations, but anchor nodes may or may not cheat in our localization model. Since we assume a distance-based localization strategy, the output of our localization algorithm can be defined by a function of measured distances from the neighbouring reference nodes: $s_{i}\leftarrow$ Locate(${\mathrm{NB}}_{i}$).

## 3. Robust and Cooperative Localization with Malicious Anchors

In this section, we describe the detailed algorithm of acoustic distance-based localization for underwater sensor networks in hostile environments. The Malicious anchors Voting-based Cooperative Localization (MVCL) algorithm is to use a few trustworthy anchor nodes with known locations mixed in malicious nodes to derive the locations of other nodes deployed in underwater region. However, malicious anchor nodes are confused with ordinary anchor nodes and hard to be distinguished. In this paper, the whole localization process is divided into two parts: malicious anchors elimination and ordinary nodes localization. In the first phase, we propose a reputation voting in 1-Hop neighbors based malicious anchor nodes detection and elimination method. In the second phase, we use an iterative localization method for each un-localized sensor nodes using honest anchor nodes and already localized sensor nodes.

As we know, accurate clock synchronization is not possible if underwater nodes belong to different clock domains. In this paper, we use an improved ToA method for ranging. Sensor node $S_{i}$ sends a ranging request packet which records transmission start time $T_{0}$. After receiving the request packet, sensor node $S_{j}$ records transmission arrival time $T_{1}$, and then sends a reply packet containing information, such as its own location, reception time, and transmission time at time $T_{2}$. Node $S_{i}$ records the time $T_{3}$ when receiving the reply packet. We can find that $T_{0}$, $T_{3}$ and $T_{1}$, $T_{2}$ belong to the clock domain of $S_{i}$ and $S_{j}$, respectively. However, no time synchronization is required between $S_{i}$ and $S_{j}$. The distance between $S_{i}$ and $S_{j}$ is calculated as follows,

(6) $$\begin{array}{c} \tilde{dst}\left(S_{i},S_{j}\right)=\frac{\left(T_{3}-T_{0}\right)-\left(T_{2}-T_{1}\right)}{2}\times c. \end{array}$$

### 3.1. Reputation Voting-Based Malicious Anchors Detection and Elimination

Due to malicious environments, we assume malicious anchor may declare a wrong location in its Hello beacon packets, or carefully manipulate anchor signals to affect the distance measurement. In this section, the proposed malicious anchors detection and elimination method is based on observation that malicious location references introduced by attacks are intended to mislead a sensor node about its location, and thus are usually inconsistent with the honest anchors. Moreover, we develop an iterative and distributed method that allows neighboring reference nodes cast reputation votes so that it can be executed in resource constrained underwater sensor nodes. Since our techniques only utilize distances measured from anchor nodes, there is no extra communication overhead involved when compared to the other localization schemes.

Intuitively, location information introduced by a malicious attack is aimed at misleading a reference node about its location. To take advantage of this observation, we use distance difference among the location references to identify and remove malicious ones. The distance difference value between measured distance and calculated distance is defined as:

(7) $$\begin{array}{c} D_{jt}\leftarrow \left|dst\left(V_{j},V_{t}\right)-\tilde{dst}\left(V_{j},V_{t}\right)\right|, \end{array}$$

where $dst(V_{j},V_{t})$ denotes Euclidean distance between $V_{j}$ and $V_{t}$, which is calculated by coordinate values declared in Hello beacon messages, and $\tilde{dst}\left(V_{j},V_{t}\right)$ denotes measured distance between $V_{j}$ and $V_{t}$ in practice, which can be calculated with Equation (6).

To harness this observation, if $D_{jt}<\eta$, it will cast a positive vote $V_{P}$, otherwise it will cast a negative vote $V_{N}$. Quite evidently, η is bounded by inconsistent malicious degree ζ. Moreover, in this paper, we select and use a simple, threshold-based confidence value to determine if the location reference is malicious or honest. The mentioned confidence value $C_{t}$ of reference node $V_{t}$ is defined as following equation,

(8) $$\begin{array}{c} C_{t}=\frac{VP_{t}+1}{VP_{t}+1+VN_{t}+1}. \end{array}$$

The reference node $V_{t}$ will be judged as a honest anchor if $C_{t}>0.5$; otherwise, it will be regarded as a malicious one. Malicious anchors can be identified and removed one by one using our predefined confidence equation, and repeat the above process until all anchor nodes have been checked.

**Figure 2:**
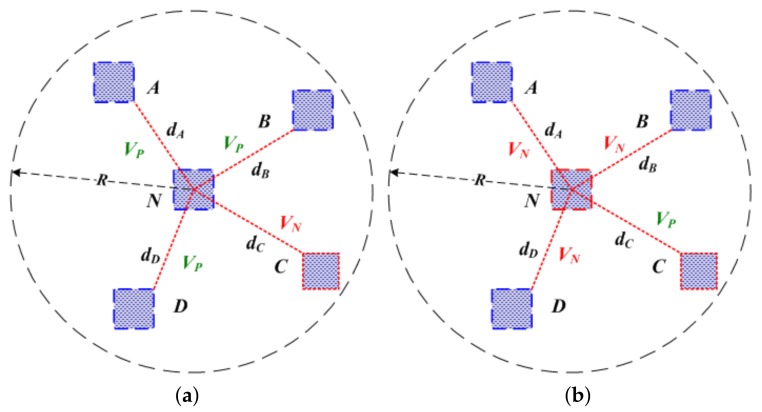
Reputation voting principle: (**a**) Case-1; (**b**) Case-2.

For example, in Case-1 of Figure 2, there are three honest anchor nodes A,B,D and one malicious anchor node *C* in the 1-Hop neighbors of anchor node *N*, that is to say, $|d_{A}-\tilde{d_{A}}|<\eta$, $|d_{B}-\tilde{d_{B}}|<\eta$, $|d_{D}-\tilde{d_{D}}|<\eta$, $|d_{C}-\tilde{d_{C}}|\geq \eta$. As a result, anchor node *N* can acquire three $V_{P}$ and one $V_{N}$ ballots from its four neighbor nodes A,B,C,D, thus C>0.5, therefore anchor node *N* will be regarded as a honest anchor node. In Case-2 of Figure 2, there are one $V_{P}$ ballot from anchor node *C* and three $V_{N}$ ballots from anchor nodes A,B,D, thus C<0.5, so anchor node *N* will be regarded as a malicious anchor node. This process will repeat until all anchor nodes have been checked, thus anchor collection A can be divided into two parts at last: malicious anchor sets M and honest anchor sets H.

### 3.2. Iterative Localization after Malicious Anchors Elimination in 3D Underwater Region

**Figure 3:**
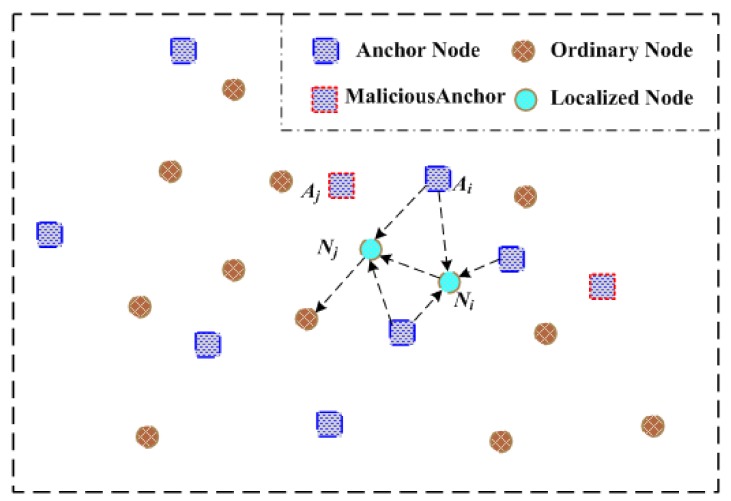
Principle of iterative localization.

After previous malicious anchors detection and elimination, we then utilize an iterative localization method within trusted reference nodes to locate the whole underwater sensor nodes. Iterative localization sequentially merges the position-known reference nodes in underwater sensor networks to locate position-unknown ordinary sensor nodes finally. The arrows shown in Figure 3 mean localization assistance direction, which are from reference nodes to nodes with unknown locations. Taking advantages of recursive location estimation procedures, a large number of un-localized sensor nodes are positioned and become reference nodes.

**Figure 4:**
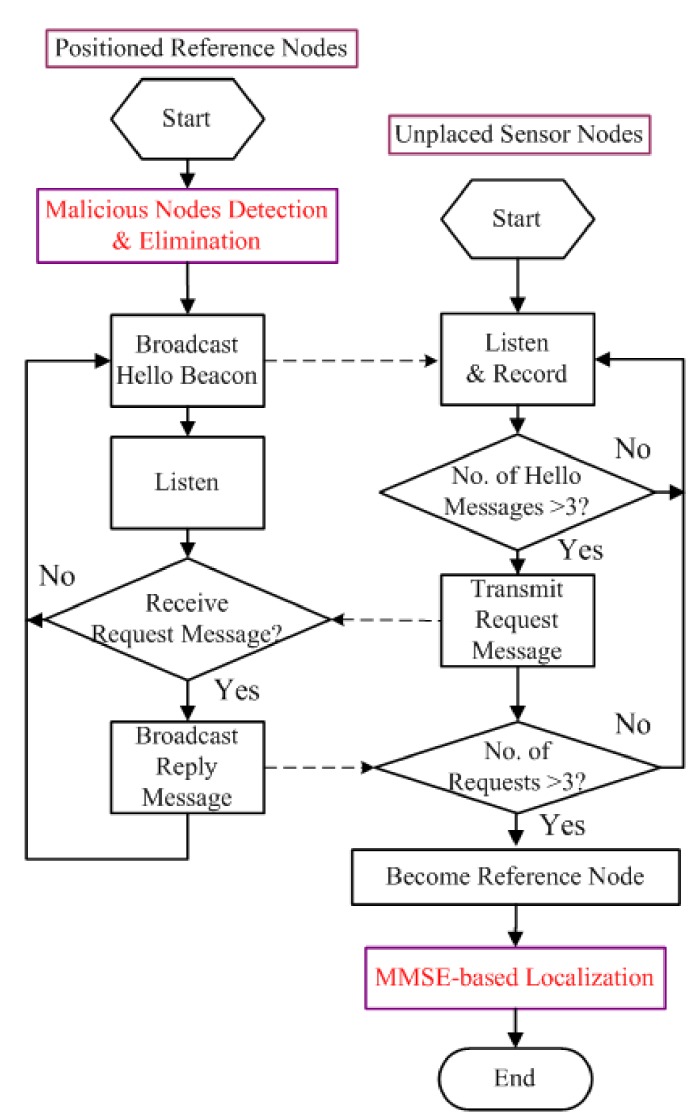
Flow chart of iterative localization method. MMSE = Minimum Mean Squared Error.

In our iterative location estimation algorithm of Figure 4, ordinary sensor nodes obtain their positions with assistance of reference nodes comprising of anchor nodes and sensor nodes having been already localized. Since MMSE (Minimum Mean Squared Error) based location calculation method [9] can deal with stochastic measurement errors better if there are more honest reference nodes, however it needs to keep as many reference nodes as possible when malicious anchors are removed. Suppose q>3 reference nodes with coordinates $\left(x_{1},y_{1},z_{1}\right),\left(x_{2},y_{2},z_{2}\right),\cdots ,\left(x_{q},y_{q},z_{q}\right)$ around certain sensor node with coordinates (x,y,z), measured relative distances between sensor node and reference nodes are $d_{1},d_{2},\cdots ,d_{q}$, respectively. Therefore, we get a series of linear equations with assumption that *q* reference nodes around this sensor node,

(9) $$\left\{\begin{array}{c} {\left(x_{1}-x\right)}^{2}+{\left(y_{1}-y\right)}^{2}+{\left(z_{1}-z\right)}^{2}=d_{1}^{2}+\omega_{1}^{2}\\ {\left(x_{2}-x\right)}^{2}+{\left(y_{2}-y\right)}^{2}+{\left(z_{2}-z\right)}^{2}=d_{2}^{2}+\omega_{2}^{2}\\ \cdots \;\cdots \\ {\left(x_{q}-x\right)}^{2}+{\left(y_{q}-y\right)}^{2}+{\left(z_{q}-z\right)}^{2}=d_{q}^{2}+\omega_{q}^{2}, \end{array}\right.$$

where $\omega_{1},\omega_{2},\cdots ,\omega_{q}$ are white Gaussian noise with unit value caused by measurements. On subtracting the last equation from the first q−1 equations set, hence we obtain,

(10) $$\begin{array}{c} PZ=B+\omega , \end{array}$$

where $Z={\left[x\;y\;z\right]}^{T}$, $\omega =[\omega_{1}^{2}\;\omega_{2}^{2}\;,\cdots ,\;\omega_{q}^{2}]$, $P=\left[\begin{array}{ccc} 2(x_{1}-x_{q}) & 2(y_{1}-y_{q}) & 2(z_{1}-z_{q})\\ 2(x_{2}-x_{q}) & 2(y_{2}-y_{q}) & 2(z_{2}-z_{q})\\ \cdots \; & \cdots \; & \cdots \\ 2(x_{q-1}-x_{q}) & 2(y_{q-1}-y_{q}) & 2(z_{q-1}-z_{q}) \end{array}\right]$ and $B=\left[\begin{array}{c} x_{1}^{2}-x_{q}^{2}+y_{1}^{2}-y_{q}^{2}+z_{1}^{2}-z_{q}^{2}+d_{1}^{2}-d_{q}^{2}\\ x_{2}^{2}-x_{q}^{2}+y_{2}^{2}-y_{q}^{2}+z_{2}^{2}-z_{q}^{2}+d_{2}^{2}-d_{q}^{2}\\ \cdots \;\cdots \\ x_{q-1}^{2}-x_{q}^{2}+y_{q-1}^{2}-y_{q}^{2}+z_{q-1}^{2}-z_{q}^{2}+d_{q-1}^{2}-d_{q}^{2} \end{array}\right]$.

As a result, the estimated position information $\hat{Z}={\left[\hat{x}\;\hat{y}\;\hat{z}\right]}^{T}$ can be obtained as,

(11) $$\begin{array}{c} \hat{Z}={\left(P^{T}P+I\omega \right)}^{-1}P^{T}B, \end{array}$$

where I is an identity matrix.

Finally, the detailed flow chart of iterative localization is described in Figure 4. Once localization process begins, each anchor node broadcasts a Hello beacon message containing its position information. After that, these sensor nodes having received Hello beacon messages from at least four reference nodes calculate their own positions using measured distances between reference nodes through ToA or TDoA method, afterwards become reference nodes. As a result, while more sensor nodes acquiring their positions and joining in reference nodes collection, the number of reference nodes increase gradually.

### 3.3. Whole Process of Iterative and Cooperative Localization Algorithm

Finally, the proposed reputation voting based cooperative localization algorithm in order to tolerate malicious anchors and improve localization robustness has the following key steps.

- Step 1: Each anchor node broadcasts Hello beacon message to its 1-Hop neighbours, which comprises of node ID, coordinates and etc. After that, distance-based acoustic ranging processes, such as ToA algorithm between itself and its 1-Hop neighboring reference nodes, are carried out to compare to calculated distance by Hello beacon messages.
- Step 2: For each anchor node, its 1-Hop neighboring reference nodes cast reputation votes according to the difference between Euclidean distance and measured distance. The voting result is that most of malicious anchor nodes can be detected and eliminated step by step.
- Step 3: After previous malicious anchors detection and elimination, an iterative localization method is applied within trusted reference nodes. Some sensor nodes become reference nodes and help to localize other sensor nodes if they are available for localization.
- Step 4: For each un-localized sensor nodes, MMSE-based localization method is applied to calculate position information. Such iterative process will not stop until all sensor nodes are checked or localized.

For clarity, the proposed MVCL algorithm is described as detailed pseudo-code in Algorithm 1. The Hello broadcast process is carried out at network initialization phase, and meanwhile each underwater node maintains its neighbor information table and perform distributed reputation voting.

**Algorithm 1** Pseudo-code for Malicious Voting-Based Cooperative Localization algorithm.
Input: X, dst(S_i,A_j),dst(A_i,A_j)∀S_i∈S,∀A_i,A_j∈AInitialization: $H\overset{A}{\leftarrow }$
   $M\overset{\emptyset }{\leftarrow }$
   $V\overset{A}{\leftarrow }$
   $VP_{i}=0,VN_{i}=0\;\forall i\in \left[1,...,v\right]$
   for i← 1 to *n* do      for j← 1 to *v* do         while $dst(S_{i},V_{j})\leq R$ do            ${\mathrm{NB}}_{i}\leftarrow {\mathrm{NB}}_{i}\cup V_{j}$
            for t← 1 to *v* do               while $dst(V_{j},V_{t})\leq R$ do                  ${\mathrm{NB}}_{i}\leftarrow {\mathrm{NB}}_{i}\cup V_{t}$
                  $D_{jt}\leftarrow dst\left(V_{j},V_{t}\right)-\tilde{dst}\left(V_{j},V_{t}\right)$
                  if $D_{jt}<\eta$ do                     $VP_{j}\leftarrow VP_{j}+1$
                  else $VN_{j}\leftarrow VN_{j}+1$
                  end if               end while            end for            $C_{j}=\frac{VP_{j}+1}{VP_{j}+1+VN_{j}+1}$
            if $C_{j}>0.5$ do                $H\leftarrow H\cup V_{t}$;    $M\leftarrow M\setminus V_{t}$
            else               $H\leftarrow H\setminus V_{t}$;    $M\leftarrow M\cup V_{t}$
               ${\mathrm{NB}}_{i}\leftarrow {\mathrm{NB}}_{i}\setminus V_{t}$
            end if         end while      end for      while $|{\mathrm{NB}}_{i}|\geq 4$ do         $S_{i}\leftarrow$ Locate(${\mathrm{NB}}_{i}$)         $V\leftarrow V\cup S_{i}$
         v=v+1
      end while   end for   Output: $\tilde{Y}$


### 3.4. Algorithm Discussions and Error Analysis

**Figure 5:**
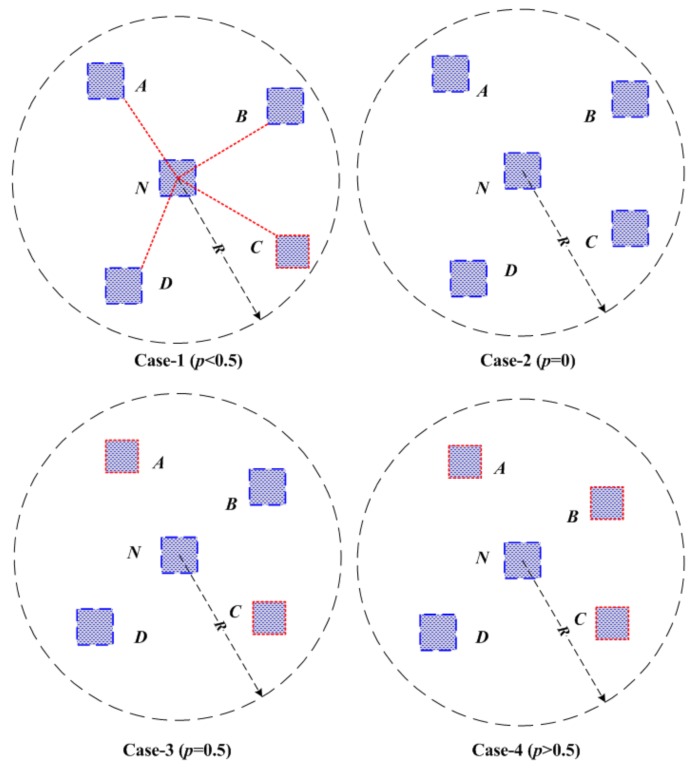
Four cases of different malicious anchor percent.

Through malicious anchors detection and elimination approach, the proposed iterative and cooperative localization method is robust to the existence of malicious anchors. However, in the presence of malicious anchor nodes, what are the necessary and sufficient conditions to guarantee a bounded error during 3-dimensional location estimation? As we know, the value of *k* clearly has a great influence on whether we can achieve a bounded localization error. Four cases of distributed reputation voting (p<0.5,p=0,p=0.5,p>0.5) are illustrated in Figure 5, where red box squares and blue box squares denote malicious anchor nodes and honest anchor nodes, respectively. It is obviously that reputation voting results are prone to be errors when p≥0.5 with our defined confidence equation. Then, we try to obtain the necessary condition for robust 3-dimensional localization in the presence of a certain amount of malicious nodes.

In the following, we will propose the lower bound theorem and moreover prove by a contradiction argument that if the numbers of malicious nodes $k\geq \frac{m-3}{2}$, the location of the unknown sensor node cannot be calculated with great accuracy by any algorithms in three-dimensional space. The necessary condition for getting a bounded localization error out of any distance-based localization algorithm is as follows.

**Theorem** **1.** *Suppose that $k\geq \frac{m-3}{2}$, then, for any distance-based localization algorithm, for any locations of anchor nodes, there exists a scenario in which localization error e is unbounded.*

**Proof.**  Without loss of generality, we assume there is an algorithm when the number of malicious nodes $k=\frac{m-3}{2}$, so that the positioning error *e* can always be smaller than *a*, where *a* is a constant, that is, *e* is bounded. We show that this assumption can lead to contradictions.

In our model, we consider a single adversary who controls all malicious anchor nodes and decides measured distance for all $A_{i}\notin H$. This is a very strong adversary model allowing malicious nodes to collude with each other so as to mislead the location. We will prove that for a fixed set of anchor nodes, and we do not know the identity of malicious nodes, if the above assumption is true, then at least two different scenarios have the same distance distribution for sensor node to be positioned. This leaves no way to distinguish between the defined two scenarios.

**Figure 6:**
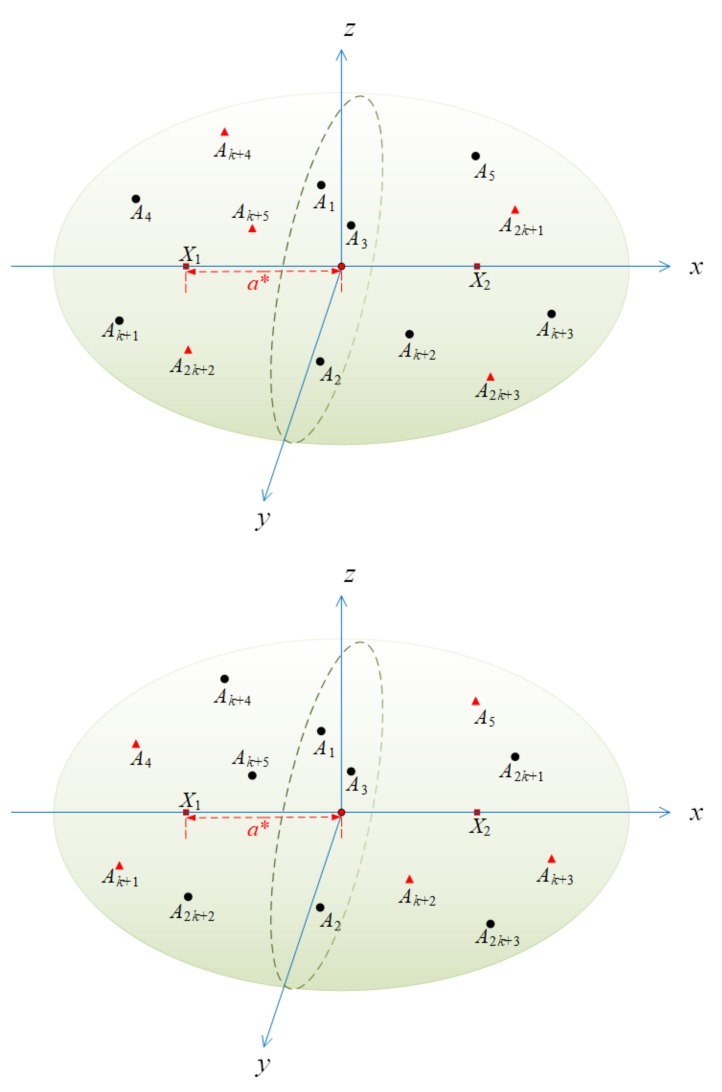
Two scenarios to prove localization bound theorem (Case-1 in upper, and Case-2 in lower).

The specific distribution of anchor nodes is as follows. Consider the two scenarios Case-1 and Case-2 in Figure 6. The positions of all anchor nodes are the same in both scenarios, but it is supposed that the set of honest anchor nodes and the set of malicious anchor nodes are different in each scene. Three anchor nodes, $A_{1}$, $A_{2}$, $A_{3}$, are selected that are not on a straight line to determine a plane.

In Case-1, select a point $X_{1}$ as the position of sensor node to be located, and make a straight line *L* through $X_{1}$ to make it perpendicular to the plane $A_{1}A_{2}A_{3}$. It should be noted that sensor node $X_{1}$ to be positioned needs to satisfy the condition that the distance $a^{*}$ between $X_{1}$ and plane $A_{1}A_{2}A_{3}$ needs to be more than the maximum positioning error *e* that we can accept, that is, $a^{*}\geq a$. In Case-2, we place sensor node to be located on the line *L* and have the same distance $a^{*}$ from plane $A_{1}A_{2}A_{3}$.

In Case-1, the set of honest anchors is $H_{1}=\left\{A_{1},A_{2},A_{3},A_{4},\ldots ,A_{k+3}\right\}$. Position-unknown node *X* is located at $X_{1}$. $\tilde{dst_{1}}\left(X,A_{i}\right)$ denotes the measurement distance $\tilde{dst}\left(X_{1},A_{i}\right)$ in scenario Case-1. In scenario Case-2, the set of honest anchors is $H_{2}=\left\{A_{1},A_{2},A_{3},A_{k+4},\ldots ,A_{2k+3}\right\}$. Position-unknown node *X* is located at $X_{2}$. $\tilde{dst_{2}}\left(X,A_{i}\right)$ denotes the measurement distance $\tilde{dst_{2}}\left(X_{2},A_{i}\right)$ in Case-2.

In Case-1, we can consider that the distance provided by our adversary model for position-unknown node *X* is the distance between each malicious node and $X_{2}$ after collusion, such that:

(12) $$\begin{array}{c} \tilde{dst_{1}}\left(X,A_{i}\right)=\tilde{dst_{2}}\left(X,A_{i}\right),\;\forall i\in \left\{k+4,...,2k+3\right\}. \end{array}$$

In other words, in Case-1, we only use the information provided by the powerful adversary to locate the position that will be calculated at $X_{2}$. In a similar way, in Case-2, the distance between malicious anchor and position-unknown *X* provided by the adversary model is actually the distance between malicious node and $X_{1}$.

(13) $$\begin{array}{c} \tilde{dst_{2}}\left(X,A_{i}\right)=\tilde{dst_{1}}\left(X,A_{i}\right),\;\forall i\in \left\{4,...,k+3\right\}. \end{array}$$

Since the line $X_{1}X_{2}$ is perpendicular to the plane $A_{1}A_{2}A_{3}$, and the line segment $X_{1}X_{2}$ is divided by the plane $A_{1}A_{2}A_{3}$ equally, it is easy to see that,

(14) $$\left\{\begin{array}{c} \tilde{dst}\left(A_{1},X_{1}\right)=\tilde{dst}\left(A_{1},X_{2}\right)\\ \tilde{dst}\left(A_{2},X_{1}\right)=\tilde{dst}\left(A_{2},X_{2}\right)\\ \tilde{dst}\left(A_{3},X_{1}\right)=\tilde{dst}\left(A_{3},X_{2}\right) \end{array}.\right.$$

In this way, we can find that $A_{1}$ and $A_{2}$ have the same distribution for $X_{1}$ and $X_{2}$,

(15) $$\left\{\begin{array}{c} \tilde{dst_{1}}\left(X,A_{1}\right)=\tilde{dst_{2}}\left(X,A_{1}\right)\\ \tilde{dst_{1}}\left(X,A_{2}\right)=\tilde{dst_{2}}\left(X,A_{2}\right)\\ \tilde{dst_{1}}\left(X,A_{3}\right)=\tilde{dst_{2}}\left(X,A_{3}\right) \end{array}.\right.$$

On the other hand, by our assumption, the output errors in both scenarios are less than *a*,

(16) $$\left\{\begin{array}{c} e=\tilde{dst}\left(X_{1},O_{1}\right)<a\\ e=\tilde{dst}\left(X_{2},O_{2}\right)<a \end{array}.\right.$$

Now, we can see that the distance provided by the same anchor node is roughly the same whether it is in Case-1 or Case-2. It is assumed that the output O of the positioning algorithm can be defined by a function F of the measured distance $\tilde{dst}\left(X,A_{i}\right)$ from the position-unknown node *X* to each anchor node $A_{i}$ in the networks, then,

In Case-1, there is,

$$O_{1}=F\left(\tilde{dst_{1}}\left(X,A_{1}\right),\tilde{dst_{1}}\left(X,A_{2}\right),...,\tilde{dst_{1}}\left(X,A_{2k+3}\right)\right).$$

In Case-2, there is,

$$O_{2}=F\left(\tilde{dst_{2}}\left(X,A_{1}\right),\tilde{dst_{2}}\left(X,A_{2}\right),...,\tilde{dst_{2}}\left(X,A_{2k+3}\right)\right).$$

According to the above inference, it can be seen that the independent variables of function F are the same in Case-1 and Case-2, so we can conclude that $O_{1}=O_{2}$.

Then, $dst\left(X_{2},O_{1}\right)=dst\left(X_{2},O_{2}\right)$. Since our previous assumption is that the output error *e* is less than the constant *a*, Consequently,

(17) $$2a^{*}=\tilde{dst}\left(X_{1},X_{2}\right)\leq \tilde{dst}\left(X_{1},O_{1}\right)+\tilde{dst}\left(X_{2},O_{1}\right)=\tilde{dst}\left(X_{1},O_{1}\right)+\tilde{dst}\left(X_{2},O_{2}\right)=e+e=2e.$$

We get the answer $a^{*}<e$, but it is contradictory to our assumption $a^{*}\geq a\geq e$. As a result, the above assumption is not true. □

On the contrary, if the number of malicious nodes *k* is smaller than $\frac{m-3}{2}$, where *m* is the number of anchor nodes providing information, then well-designed localization algorithms may provide certain localization accuracy. The following simulation results of our proposed MVCL algorithm verify this conclusion.

## 4. Simulation Results

**Figure 7:**
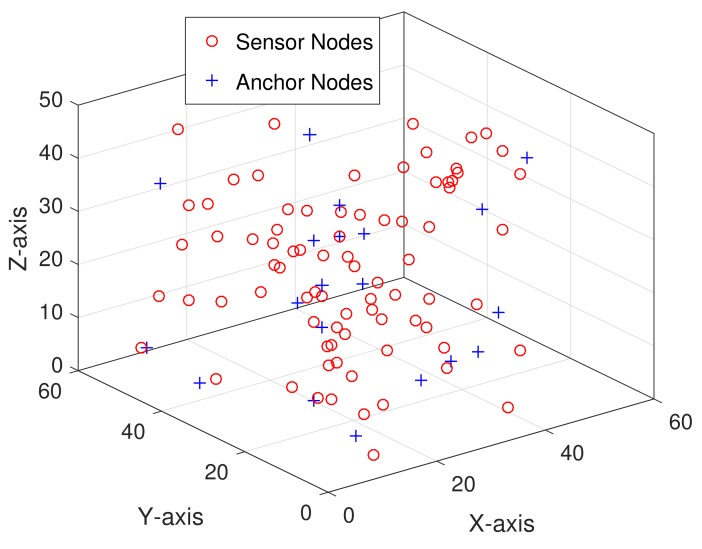
Simulation topology snapshot (n=80,m=20).

**Figure 8:**
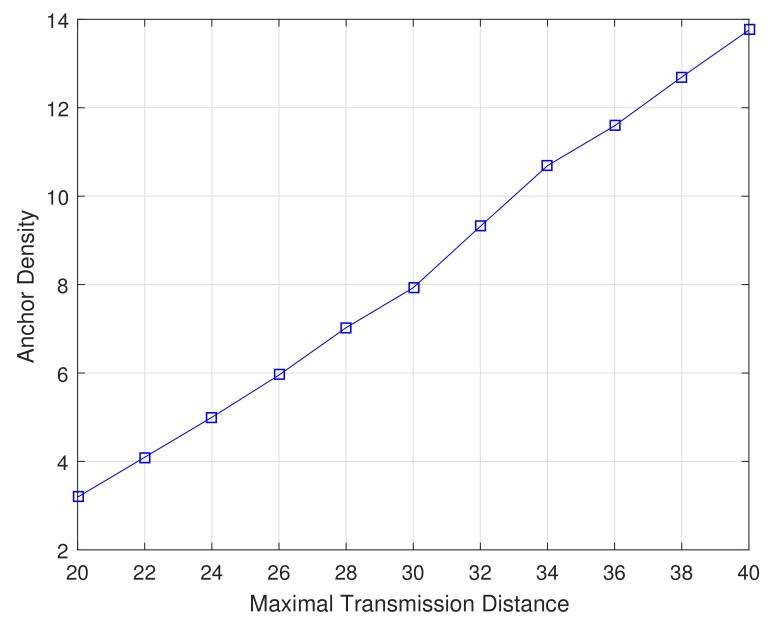
Anchor density with transmission range (n=80,m=20).

In this section, we compare the localization performance of proposed algorithms with that of MMSE [9] and ARMMSE [24] through simulation experiments. Simulation is set up using 100 nodes with typical case of 80 sensors and 20 anchors randomly distributed in a cube of 50×50×50 unit ${}^{3}$. The MATLAB 2016b simulator is used as simulation tool. Figure 7 depicts an initial deployment snapshot of our simulation scenario. The maximal transmission distance *R* is determined following appropriate anchor density function from 4 to 12, which is shown in Figure 8. The anchor density is defined as the average number of neighboring nodes. All simulation processes are repeated for 100 Monte Carlo runs to obtain average results in this paper. Performance of the proposed MVCL algorithm is mainly evaluated in terms of average localization error and localization coverage ratio. Average localization error means the difference value between actual position values and estimated position values. Localization coverage ratio denotes that the percent between the number of localized nodes and all sensor nodes at certain percent ratio of anchor nodes. Obviously, localization coverage ratio will increase when the number of anchor nodes and sensor nodes increases. In order to make simulation setup and results clear and easy to understand, we list some of main parameters for simulation in Table 2.

**Figure 9:**
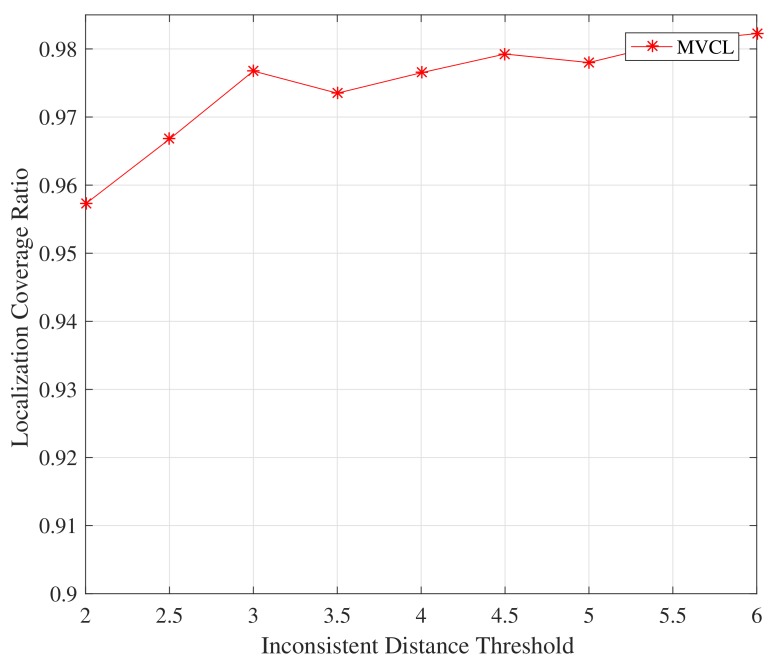
Localization coverage with inconsistent distance threshold η (n=80,m=20,k=4,R=30, ζ=15).

**Figure 10:**
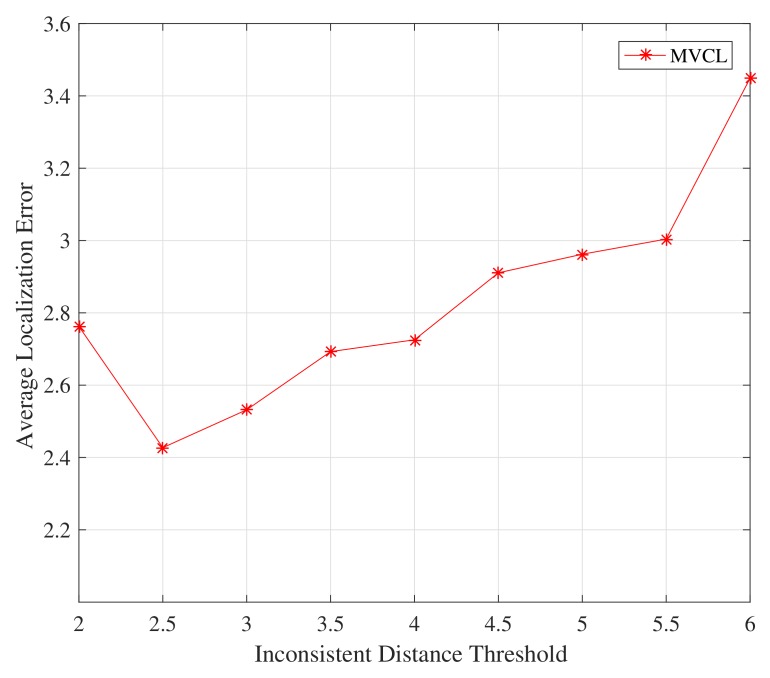
Localization error with inconsistent distance threshold η (n=80,m=20,k=4, R=30, ζ=15).

Firstly, we study the impact on the proposed MVCL algorithm by inconsistent distance threshold η. Figure 9 and Figure 10 depict the relations between localization performance and inconsistent distance threshold η. It is apparent from Figure 9 and Figure 10 that the localization coverage and localization error will increase with much larger inconsistent distance threshold. It should not be hard to understand since much more anchors are identified and removed if η is relatively small. However, this effect is not very obvious, so we set η=3 in the following simulations.

**Figure 11:**
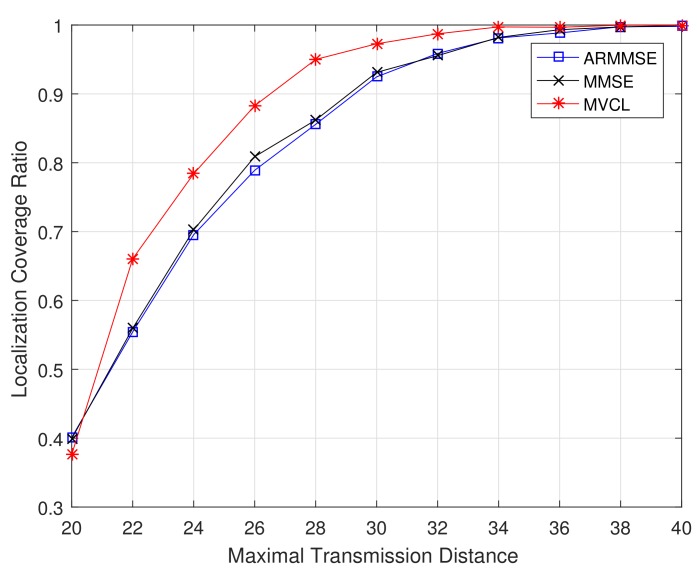
Localization coverage ratio with transmission range *R* (n=80,m=20,k=4,ζ=15,η=3).

**Figure 12:**
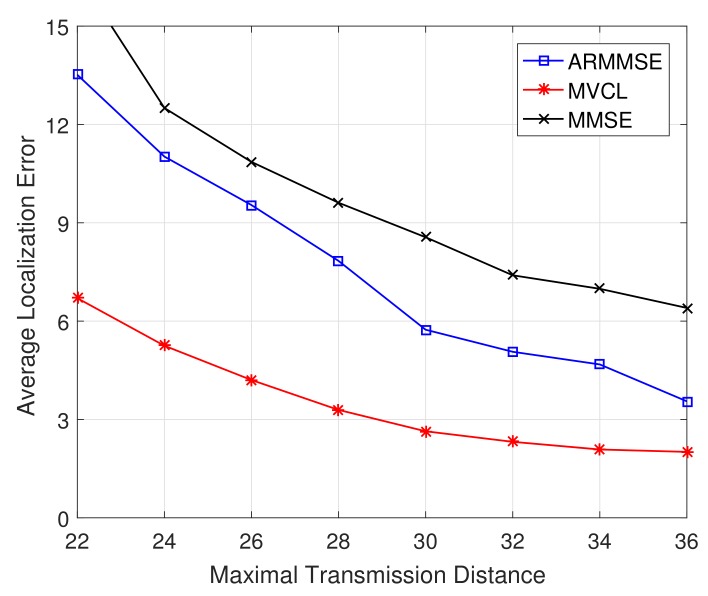
Average localization error with transmission range *R* (n=80,m=20,k=4,ζ=15,η=3).

Subsequently, we got comparable results with related methods, respectively. Figure 11 and Figure 12 illustrate the localization coverage ratio and average localization error varying with maximal transmission distance *R*, respectively. It can be shown that localization coverage ratio of MVCL algorithm is much larger than that of MMSE and ARMMSE, while average localization error of MVCL is much smaller than that of MMSE and ARMMSE. This is because MMSE does not consider the impacts by malicious anchors, which would reduce localization accuracy. Furthermore, the malicious exclusion rule of ARMMSE is so strict that it is not robust enough for malicious anchors.

**Figure 13:**
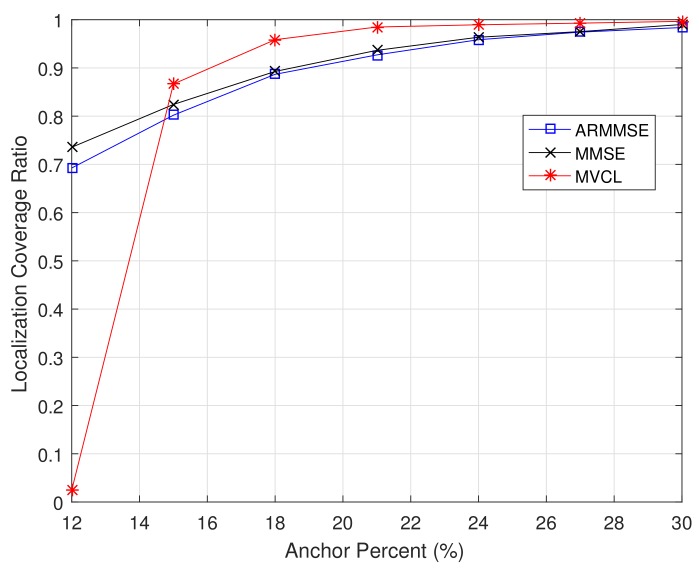
Localization coverage ratio with anchor percent (n+m=100,m=1230,R=30,k=4, ζ=15,η=3).

**Figure 14:**
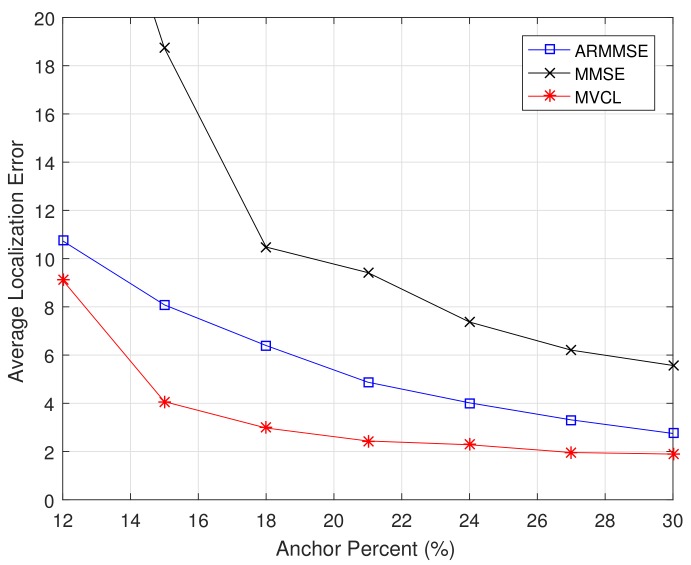
Average localization error with anchor percent (n+m=100,m=1230,R=30,k=4, ζ=15,η=3).

Moreover, we compare localization performance with different anchor percents. Figure 13 and Figure 14 illustrate the localization coverage ratio and average localization error varying with anchor percent $\frac{m}{m+n}$, respectively. It can be concluded from results that localization coverage ratio of MVCL is larger than that of MMSE and ARMMSE along with different anchor percents, while average localization error of MVCL is much smaller than that of MMSE and ARMMSE under different anchor percents. It is worth noting that the localization coverage ratio is relatively lower of MVCL algorithm when anchor percent $\frac{m}{m+n}<15\%$. The reason lies in the number of malicious anchors is relatively higher under that condition when k=4, so as to bring down localization coverage ratio. As a result, simulation results verify that our MVCL algorithm can handle such networks case in the existence of malicious anchors.

**Figure 15:**
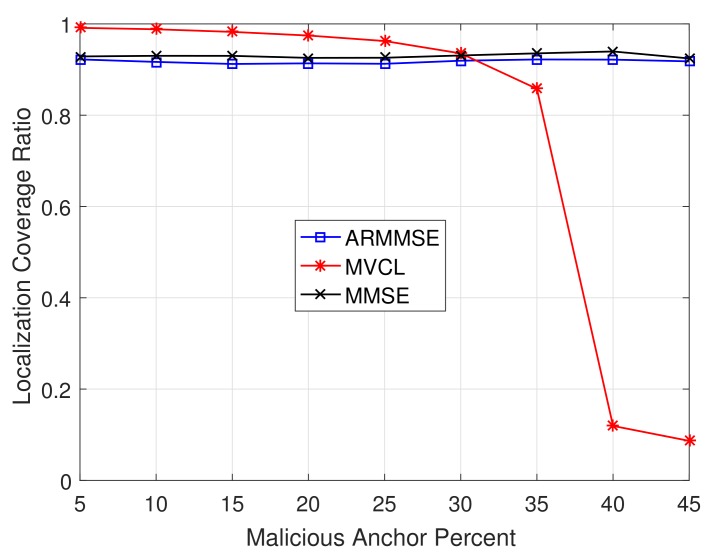
Localization coverage ratio with malicious anchor percent *p* (n=80,m=20,R=30,ζ=15, η=3).

**Figure 16:**
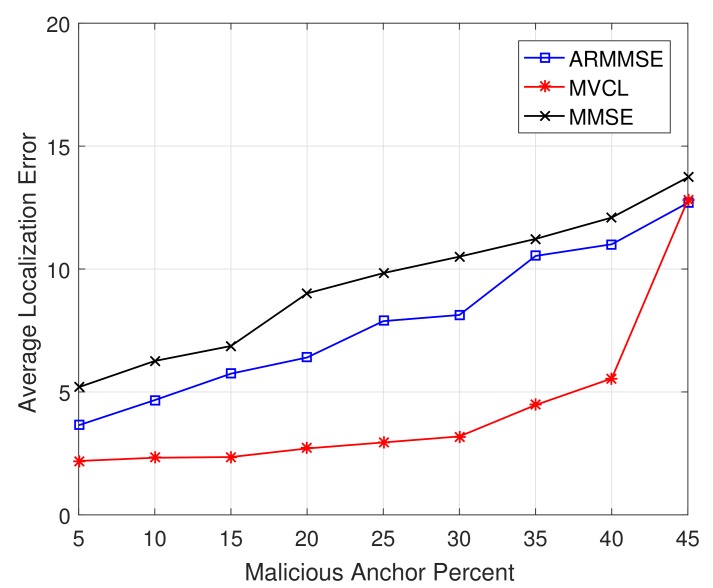
Average localization error with malicious anchor percent *p* (n=80,m=20,R=30,ζ=15, η=3).

In addition, we study the relations between localization performance and malicious anchor percent *p*. Figure 15 and Figure 16 illustrate the localization coverage ratio and average localization error varying with malicious anchor percent *p*, respectively. It shows that localization coverage ratio of MVCL is larger than that of MMSE and ARMMSE when p<45%, while average localization error of MVCL is much smaller than that of MMSE and ARMMSE. However, it is noted that the localization coverage ratio will have a sharp decline when p≥40% and the average localization error will increase at that time. Therefore, we verify that the proposed algorithm cannot handle such a case with nearly half malicious anchors. Moreover, the above result tallies with the necessary and sufficient conditions to guarantee a bounded localization error.

**Figure 17:**
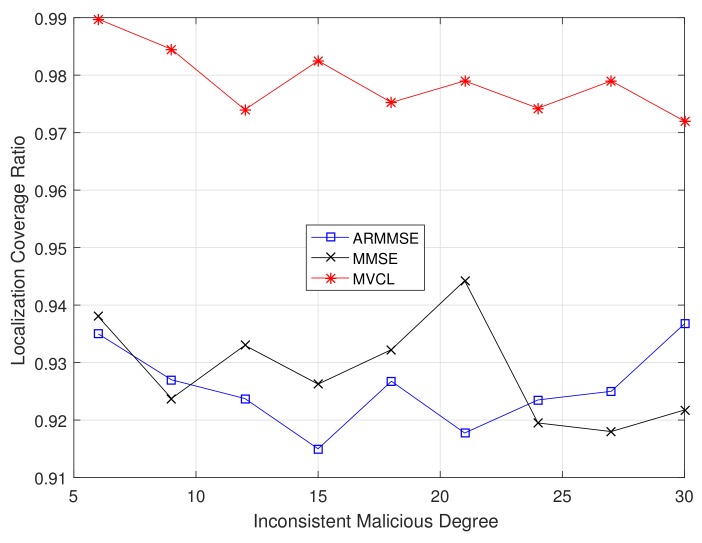
Localization coverage ratio with inconsistent malicious degree ζ (n=80,m=20, R=30, k=4,η=3).

**Figure 18:**
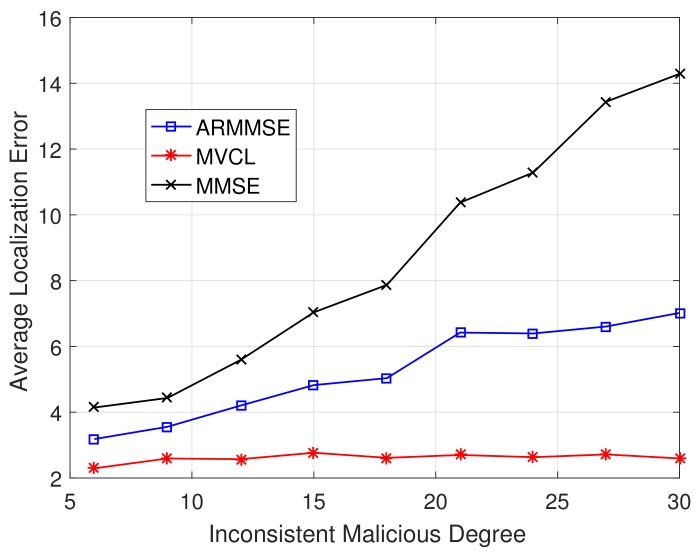
Average localization error with inconsistent malicious degree ζ (n=80,m=20, R=30, k=4,η=3).

Besides, the relations between localization performance and inconsistent malicious degree are verified. Figure 17 and Figure 18 illustrate localization coverage ratio and average localization error varying with inconsistent malicious degree ζ, respectively. It is clearly that localization coverage ratio of MVCL is larger than that of MMSE and ARMMSE, while average localization error of MVCL is much smaller than that of MMSE and ARMMSE at different ζ. In addition, the performance improvement advantages are more obvious when ζ is larger. In most cases, our proposed MVCL algorithm can outperform MMSE and ARMMSE roughly two-fold.

**Figure 19:**
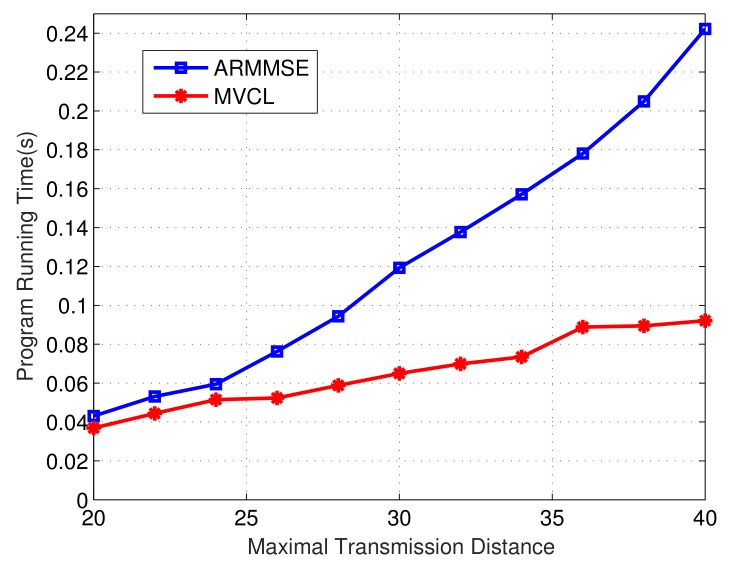
Operation time with maximal transmission distance (n=80,m=20,k=4,ζ=15,η=3).

**Figure 20:**
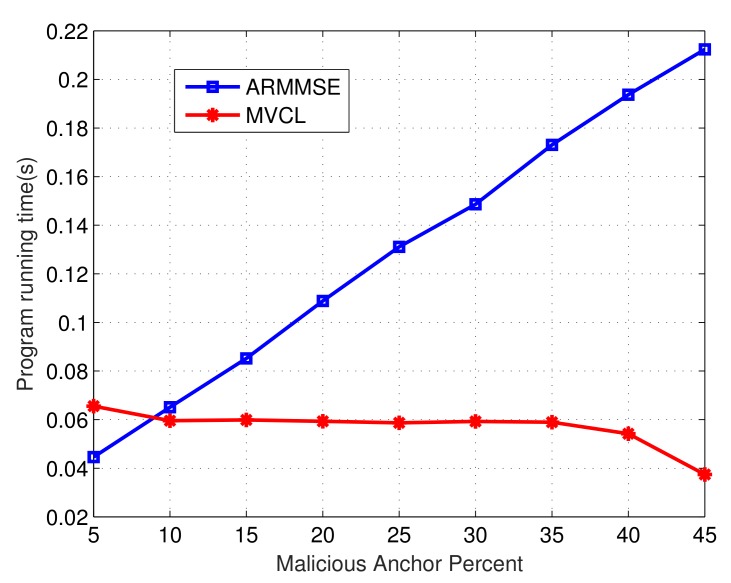
Operation time with malicious anchor percent *p* (n=80,m=20,R=30,k=4,ζ=15, η=3).

Finally, we compare the program operation time of our MVCL algorithm to that of ARMMSE. Figure 19 and Figure 20 denote the change of total running time of localization algorithms with the change of maximal transmission distance and malicious anchor percent, respectively. It becomes apparent that the running time of MVCL algorithm outperform that of ARMMSE. Going furth, unlike the existing ARMMSE algorithm, the program running time of our MVCL algorithm will not increase with larger malicious anchor percent. Hence, it is very suitable for large-scale underwater sensor networks with this excellent feature.

Summarize the work, in all the four test cases, localization coverage ratio and average localization error of MVCL outperform that of MMSE and ARMMSE. The derived good performance is because: (1) we use a distributed reputation voting idea within neighboring reference nodes to eliminate the impacts by malicious anchor nodes; and (2) we use MMSE based iterative location estimation to determine accurate location information with more reference nodes, so as to improve localization robustness.

## 5. Conclusions

In many military applications of underwater sensor networks in the existence of malicious anchor nodes, it is crucial to determine the accurate location of sensor nodes. For this matter, this paper investigated an iterative and cooperative localization algorithm for three-dimensional underwater sensor networks. The proposed MVCL algorithm was based on distributed reputation voting and cooperative iteration to identify and remove malicious anchors, so as to guarantee its robustness and effectiveness. More precisely, we analyzed the necessary condition to guarantee a bounded error in the presence of certain number of malicious anchors. Extensive simulation results verify that the proposed localization algorithm is more efficient than existing algorithms. In our future works, the dynamics of sensor nodes and anchor nodes will be considered.

## Figures and Tables

**Table 1:** List of Notations.

Notation	Definition
S	set of sensor nodes
A	set of anchor nodes
V	set of reference nodes
*n*	number of sensor nodes
*m*	number of anchor nodes
*v*	number of reference nodes
$S_{i}$	*i*-th sensor node, i=1,...,n
$A_{j}$	*j*-th anchor node, j=1,...,m
$V_{t}$	*t*-th reference node, t=1,...,v
$C_{t}$	confidence value of $V_{t}$
$Y=[s_{1},s_{2},...,s_{n}]$	coordinates set for sensor nodes
$s_{i}=<x_{i},y_{i},z_{i}>$	coordinates of sensor node $S_{i}$
$\tilde{s_{i}}=<\tilde{x_{i}},\tilde{y_{i}},\tilde{z_{i}}>$	measured coordinates of sensor node $S_{i}$
$\tilde{Y}=\left[\tilde{s_{1}},\tilde{s_{2}},...,\tilde{s_{n}}\right]$	measured coordinates of sensor nodes
$X=[a_{1},a_{2},...,a_{m}]$	coordinates set for anchor nodes
$a_{j}=<x_{j},y_{j},z_{j}>$	coordinates of anchor node $A_{j}$
H	set of honest anchor nodes
M	set of malicious anchor nodes
*k*	number of malicious anchor nodes
*p*	percent of malicious anchor nodes
*R*	maximal transmission distance
$dst(V_{i},V_{j})$	Euclidean distance between $V_{i}$ and $V_{j}$
$\tilde{dst}\left(V_{i},V_{j}\right)$	measured distance between $V_{i}$ and $V_{j}$
${\mathrm{NB}}_{i}$	neighboring reference nodes of sensor node $S_{i}$
$VP_{t}$	positive voting number for reference node $V_{t}$
$VN_{t}$	negative voting number for reference node $V_{t}$
$V_{P}$	positive voting
$V_{N}$	negative voting
ζ	inconsistent malicious degree
η	inconsistent distance threshold
ϵ	a small constant
δ	average localization error
*c*	underwater acoustic velocity
$\mu_{ij}$	Gaussian random variables
$\sigma^{2}$	variance of $\mu_{ij}$

**Table 2:** Simulation Notations.

Notation	Value
*n*	80
*m*	20
*k*	1–9
η	2–6
ζ	6–30
*R*	20–40
*p*	5–45%
